# Targeting mitochondrial dysfunction with traditional Chinese medicine for inflammatory bowel disease: a comprehensive review

**DOI:** 10.3389/fphar.2026.1851055

**Published:** 2026-07-09

**Authors:** Linxi Zeng, Yao Huang, Jiayi Ke, Jiaqi Han, Wenda Xie, Zhishan Ding, Fangmei Zhou

**Affiliations:** School of Medical Technology and Information Engineering, Zhejiang Chinese Medical University, Hangzhou, Zhejiang, China

**Keywords:** Crohn’s disease, inflammatory bowel disease, mitochondrial dysfunction, traditional Chinese medicine, ulcerative colitis

## Abstract

Inflammatory Bowel Disease (IBD) is a chronic relapsing inflammatory condition of the intestine, characterized by symptoms such as chronic diarrhea, abdominal pain, and weight loss. The pathogenesis of IBD is complex, with mitochondrial dysfunction being considered a key factor in its onset, progression, and persistence. Mitochondria, as the primary energy suppliers within cells, not only produce adenosine triphosphate (ATP) but also play crucial roles in regulating cellular metabolism, maintaining redox balance, and controlling cell death processes. Targeting mitochondria to regulate mitochondrial functions, including energy metabolism, oxidative stress, mitophagy, dynamics, and biogenesis, as well as maintaining the dynamic balance of the “gut microbiota-mitochondria axis,” has emerged as a promising strategy for the prevention and treatment of IBD. Traditional Chinese Medicine (TCM) has shown potential multi-target regulatory properties in preclinical studies; however, robust clinical validation and target-specific pharmacological evidence remain limited. This review explores the mechanisms and underlying connections between mitochondrial dysfunction and IBD, summarizing the current research on how active metabolites of TCM modulate mitochondrial dysfunction in the prevention and treatment of IBD. It provides new insights into the pathogenesis of the disease and opens up new avenues and strategies for the prevention, treatment, and research on IBD treatment with TCM.

## 1 Introduction

Inflammatory Bowel Disease (IBD) is a chronic relapsing inflammatory condition of the intestine, encompassing Ulcerative Colitis (UC) and Crohn’s Disease (CD). It is typically characterized by chronic diarrhea, abdominal pain, weight loss, nausea, occasional chills, and fever (Bruner et al., 2023). UC predominantly affects the colon and rectum, with lesions primarily involving the mucosa and submucosa, without extending beyond the intestinal wall. The common symptoms include abdominal pain and purulent blood-stained stools; whereas CD can affect any part of the digestive tract, although it most commonly involves the ileum or the initial part of the colon. Unlike UC, CD lesions are transmural and exhibit a segmental pattern, and the chronic inflammatory process may ultimately lead to intestinal obstruction (Khor et al., 2011; Rogler et al., 2018). A key feature of IBD is repeated damage to the intestinal epithelium, accompanied by impaired intestinal barrier function. Clinically, it is characterized by alternating phases of flare-ups, remission, and relapse (Henderson et al., 2011; Le Berre et al., 2023). In China, the annual incidence of IBD is approximately 11 cases per 100,000 people (Kaplan and Windsor, 2021), with a rising incidence in recent years (Eisenstein, 2018). A study involving 1,139 Chinese IBD patients found that 58% of patients experienced relapses during follow-up, and the overall surgical rate was 8.6%, significantly affecting patients’ health and quality of life (Liu B. H. et al., 2024). The pathogenesis of IBD remains unclear, but it may involve complex interactions between genetic, immune, microbiological, and environmental factors (Porter et al., 2020). Currently, clinical treatments primarily aim to control excessive immune responses and inflammation to promote mucosal healing, thereby alleviating and managing IBD symptoms. However, they do not cure the disease, and maintenance therapy is often required (Rutgeerts et al., 2007). Research has shown that IBD patients frequently exhibit mitochondrial dysfunction, which not only contributes to the initial onset of IBD but also plays a critical role in the persistence and relapse of the disease (Novak and Mollen, 2015; Alula et al., 2021; Ho and Theiss, 2022).

Currently, IBD treatment drugs have been successful in repairing the mucosal barrier, but they have not completely corrected the inherent mitochondrial dysfunction of intestinal epithelial cells. These abnormalities further exacerbate damage to the intestinal mucosal epithelial cells and inflammatory responses, leading to an energy crisis, oxidative stress, and latent inflammation, which weakens the barrier (Novak and Mollen, 2015; Haque et al., 2024), ultimately resulting in disease relapse. Therefore, mitochondrial dysfunction may represent an important contributor to disease relapse, and targeting mitochondrial dysfunction may provide a promising complementary therapeutic strategy for IBD, providing an advantage over traditional therapeutic approaches. Mitochondria are essential organelles within cells, playing a crucial role in cellular energy metabolism, regulation of cell survival, and apoptosis. They supply energy via oxidative phosphorylation and the TCA cycle, regulate the balance of reactive oxygen species (ROS) via antioxidant systems; selectively eliminate dysfunctional mitochondria through mitophagy, remodel the mitochondrial network through continuous fusion and fission (i.e., mitochondrial dynamics); and synthesize new mitochondria through biogenesis to replenish the mitochondrial pool. This mitochondrial homeostasis, maintained through multiple mechanisms, is vital for cell survival. Therefore, this paper will analyze the impact and mechanisms of mitochondrial dysfunction on the progression of IBD and summarize the research progress on how various active metabolites of traditional Chinese medicine (TCM) regulate mitochondrial function in the prevention and treatment of IBD.

## 2 Literature search strategy

### 2.1 Search strategy

A comprehensive literature search was conducted using PubMed and Web of Science databases for studies published up to January 2026. The search terms included combinations of “inflammatory bowel disease”, “ulcerative colitis”, “Crohn’s disease”, “mitochondria”, “mitophagy”, “oxidative stress”, “traditional Chinese medicine”, “natural products”, and “phytochemicals”. Boolean operators (“AND”, “OR”) were applied to optimize the search strategy. Both preclinical and clinical studies investigating the effects of active metabolites of TCM on mitochondrial dysfunction in IBD were included. Non-English publications, conference abstracts, duplicate studies, and studies lacking sufficient methodological details were excluded.

### 2.2 Taxonomic validation

Scientific names and taxonomic information of medicinal plants mentioned in this review were verified using authoritative botanical databases, including Plants of the World Online (POWO) and World Flora Online (WFO). Synonyms and historical nomenclature inconsistencies were manually cross-checked to ensure taxonomic accuracy and avoid ambiguity in species identification. Botanical names were updated according to currently accepted taxonomic standards where applicable.

### 2.3 Quality assessment of included studies

Most of the available evidence originated from preclinical investigations using DSS- or TNBS-induced colitis models, whereas clinical studies remained limited. Although many studies demonstrated beneficial effects on mitochondrial function, only a minority reported randomization procedures, blinding strategies, sample size calculations, or pharmacokinetic validation. Furthermore, direct measurements of mitochondrial bioenergetics, respiratory chain activity, and mitochondrial quality control pathways were inconsistently performed. Consequently, the overall strength of evidence supporting mitochondrial-targeted therapeutic effects of active metabolites from TCM in IBD should be considered moderate, and further rigorously designed mechanistic and clinical studies are required.

## 3 Mechanisms and effects of mitochondrial dysfunction on IBD progression

Mitochondrial swelling and disruption of cristae structure are commonly observed in both IBD animal models and patient tissues, indicating mitochondrial dysfunction. These changes can be detected even before the onset of inflammation during the progression of IBD (Hsieh et al., 2006). Studies have shown that in wild-type mice treated with dextran sulfate sodium (DSS), mitochondrial changes in colonic epithelial cells, including swelling, reduced ATP production, increased ROS generation, and upregulation of antioxidant proteins, occur within less than 3 days of treatment. After 7 days, tissue damage and cytokine induction are observed (Xue et al., 2017). Intestinal mucosal barrier dysfunction is a key factor in the onset and progression of IBD. Mitochondrial dysfunction leads to reduced expression of tight junction proteins, increased intestinal permeability, and the entry of pathogenic antigens into the intestinal mucosal layer, ultimately resulting in intestinal mucosal inflammation. Under chronic inflammation conditions, excessive cytokine stimulation can damage the mucus layer on the mucosal surface, making the intestinal mucosa more vulnerable to various pathogenic factors. This, in turn, further exacerbates the dysfunction of the intestinal barrier, leading to chronic colonic inflammation (Shen et al., 2009; Zorov et al., 2014; Han et al., 2020; Khaloian et al., 2020; Ray, 2020).

At the same time, the functional status of mitochondria also influences the differentiation of intestinal stem cells into various epithelial cell types, such as Paneth cells and goblet cells. Mitochondrial dysfunction is associated with abnormal Paneth cells and weakened mucosal defense in IBD (Adolph et al., 2013). Mitochondrial dysfunction—manifested as defects in oxidative phosphorylation, decreased membrane potential, increased levels of ROS, mitophagy disruption, abnormal fusion and fission, and mitochondrial DNA (mtDNA) abnormalities—leads to a dynamic imbalance in the “gut microbiota-mitochondria axis.” Through multidimensional synergistic effects, this imbalance induces intestinal inflammation and disrupts the integrity of the intestinal epithelial barrier, serving as a critical driver of IBD pathogenesis. These mechanisms are often interconnected, forming a vicious cycle. For instance, defects in oxidative phosphorylation trigger ROS bursts, which not only directly damage the intestinal epithelium but also activate inflammatory pathways, further aggravating mitochondrial and tissue damage. Mitophagy, to some extent, can inhibit inflammasome activation and alleviate disease progression.

### 3.1 IBD and mitochondrial energy metabolism abnormalities

Mitochondrial dysfunction-induced ATP depletion can impair the intestinal mucosal barrier, leading to the onset of IBD (Ruiz et al., 2020), whereas an increase in intestinal ATP levels can lead to a dramatic increase in the intestinal epithelial turnover rate (Bär et al., 2013). Intestinal epithelial cells are high-energy-demand cells, and the maintenance of the intestinal mucosal barrier integrity heavily depends on energy metabolism. ATP is the direct source of energy for intestinal mucosal epithelial cells, which generate ATP through glycolysis, the tricarboxylic acid cycle, and mitochondrial oxidative phosphorylation. Energy production in eukaryotic cells primarily occurs in the mitochondria, where the key processes are the electron transport chain and oxidative phosphorylation. The electron transport chain consists of five respiratory complexes embedded in the mitochondrial membrane: Complex I (NADH-ubiquinone reductase), Complex II (succinate-ubiquinone reductase), Complex III (ubiquinone-cytochrome c reductase), Complex IV (cytochrome c oxidase), Complex V (ATP synthase), as well as ubiquinone and cytochrome c. These complexes transfer electrons and pump protons from the mitochondrial matrix to the intermembrane space, establishing an electrochemical gradient that drives ATP synthase to transport protons, ultimately leading to ATP production (Murphy, 2009; Dixon and Stockwell, 2014). Heller et al. (2017) found that in colonic cells lacking mtDNA and in human IBD tissues, interleukin (IL) levels were elevated, and IL-8 could sense the reduction in ATP levels, regulate energy production, and play a crucial role in intestinal inflammation. The authors speculated that intestinal inflammation is promoted by reduced mitochondrial energy production.

**FIGURE 1:**
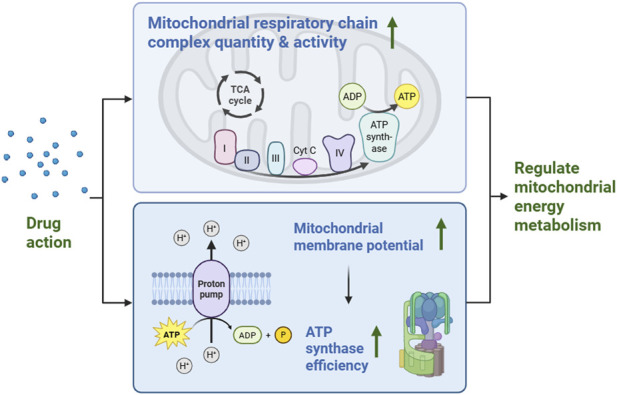
Regulating mitochondrial energy metabolism to repair damage to the intestinal mucosal barrier may be a key therapeutic approach for IBD (Zhang et al., 2024). The schematic illustrates the potential therapeutic mechanism by which drug intervention restores intestinal mucosal barrier function through modulation of mitochondrial energy metabolism. Drug action enhances the quantity and activity of mitochondrial respiratory chain complexes, thereby increasing ATP production. Simultaneously, regulation of proton pump activity and ATP synthase function contributes to the maintenance of mitochondrial membrane potential and optimization of ATP synthase efficiency. Collectively, these processes improve mitochondrial bioenergetic homeostasis and may facilitate intestinal epithelial repair, barrier integrity maintenance, and attenuation of IBD-associated mucosal injury. Upward arrows indicate enhanced activity or function. Abbreviations: ADP, Adenosine Diphosphate; ATP, Adenosine Triphosphate; Cyt C, Cytochrome C; IBD, Inflammatory Bowel Disease; TCA cycle, Tricarboxylic Acid Cycle. Created in BioRender. Zeng, L. (2026) https://BioRender.com/evtf1xr.

Mitochondrial membrane potential refers to the transmembrane voltage difference generated by the varying ion concentrations on either side of the mitochondrial inner membrane. Along with the proton gradient, the mitochondrial membrane potential forms the electrochemical gradient of hydrogen ions, which is the driving force for ATP synthesis and release, and is a sensitive indicator of mitochondrial function (Zorova et al., 2018). Clinical studies (Schürmann et al., 1999) show that ATP levels are reduced in the intestines of IBD patients, with energy levels in intestinal cells being lower than those in healthy subjects. Therefore, mitochondrial energy metabolism abnormalities may be an important factor in the pathogenesis and progression of IBD (Figure 1).

#### 3.1.1 Abnormal activity of mitochondrial respiratory chain complexes

The electron transport chain is the material basis for mitochondrial oxidative phosphorylation, which generates ATP (Vercellino and Sazanov, 2022). Therefore, a decrease in the activity of the respiratory chain complexes can hinder the electron transfer in the chain, affecting ATP production. Research has shown that in colitis mice with low mitochondrial respiratory chain complex activity and ATP levels in the intestinal mucosa, the mucosal inflammation is more severe (Bär et al., 2013). Clinical studies have indicated that in UC patients, ATP content and the activity of mitochondrial respiratory chain complex I in the affected intestinal mucosa are significantly decreased. The activity of complex II in the colon biopsy samples from UC patients is significantly lower compared to healthy controls, and the activity of complex II in normal mucosa is also reduced. In UC patients, the activities of complexes II, III, and IV in the colonic mucosa are reduced by approximately 50%-60% (Sifroni et al., 2010; Santhanam et al., 2012). Additionally, clinical case reports have shown that in an 8-year-old CD patient, mitochondrial function assessments of muscle samples revealed defects in complexes III and IV(Wang et al., 2007). Therefore, the reduction in the activity of mitochondrial respiratory chain complexes, leading to mitochondrial energy metabolism dysfunction, may be an important pathogenic mechanism in IBD.

#### 3.1.2 Abnormalities in mitochondrial transmembrane potential

Damage to the mitochondrial membrane potential leads to its decrease. Changes in mitochondrial membrane potential represent the initial stage of mitochondrial damage, directly affecting the energy that drives ATP synthesis, which in turn impacts the motility of the affected tissues (Sanderson et al., 2013). Gastrointestinal smooth muscle movement relies on the maintenance of the mitochondrial membrane potential to produce energy. Abnormalities in the mitochondrial membrane potential can lead to gastrointestinal motility dysfunction, which, according to TCM, is closely related to the onset and regulation of IBD (Zhihua, 2014). Additionally, studies have confirmed that in cellular and animal models, IND nanoparticles (IND-NPs) can restore mitochondrial dysfunction induced by dextran sulfate sodium (DSS) by increasing mitochondrial membrane potential and ATP levels (Wu P. et al., 2023). In human epithelial cells, genetic ablation of ATF7 or PINK1 can disrupt the mitochondrial membrane potential, leading to exacerbated epithelial damage, increased cytokine production, and activation of inflammatory signaling pathways such as TNF-α/NF-κB, thereby aggravating DSS-induced colitis (Liu F. et al., 2025). Therefore, the insufficient mitochondrial energy production caused by a decrease in the mitochondrial membrane potential may be an important cause of IBD.

### 3.2 IBD and mitochondrial oxidative stress

Mitochondria are the primary sites within cells where ROS are produced. Under physiological conditions, electron leakage occurs at mitochondrial respiratory chain complexes I, II, and III, where they react with oxygen and are reduced to form ROS. Due to the presence of the mitochondrial antioxidant system, the production and clearance of ROS are in dynamic balance under normal conditions (Chen et al., 2024). However, during inflammation, this balance is disrupted, leading to an imbalance between oxidation and antioxidation systems. As a result, ROS accumulate in the body, triggering oxidative stress. Under oxidative stress, ROS can act back on the mitochondria, downregulating the activity of mitochondrial respiratory chain complexes (Murphy, 2009), causing changes in mitochondrial membrane permeability (Ma et al., 2011), generating free radicals by binding with iron (Dixon and Stockwell, 2014), damaging mtDNA (Mikhed et al., 2015), affecting ATP synthesis, and disrupting mitochondrial structure, leading to mitochondrial dysfunction (Zorov et al., 2014). ROS can also attack macromolecules such as cellular proteins, nucleic acids, and lipids (Bergamini et al., 2004), causing oxidative damage to cells and disrupting cell structure. At the same time, ROS stimulate cytokine expression, activate mitochondria-dependent apoptotic pathways, and induce cell apoptosis (Circu and Aw, 2010). When mitochondria are damaged, respiratory chain dysfunction occurs, electron transfer is obstructed, electron leakage increases, and ROS generation escalates. This further damages mitochondrial structure and function, forming a vicious cycle (Moon et al., 2020).

**FIGURE 2:**
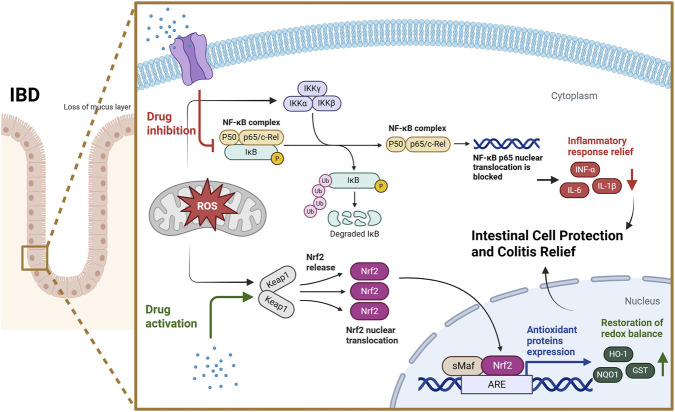
Regulating mitochondrial oxidative stress represents a key therapeutic strategy in IBD management (Dixon et al., 2012; Zhuge et al., 2023). The figure illustrates the proposed mechanism by which drug intervention suppresses oxidative stress-associated inflammation in IBD. ROS activate the NF-κB signaling pathway through IκB phosphorylation and degradation, promoting transcription of pro-inflammatory cytokines, including TNF-α, IL-6, and IL-1β. Meanwhile, drug activation of the Keap1/Nrf2/ARE pathway promotes Nrf2 nuclear translocation and antioxidant protein expression, including HO-1, NQO1, and GST, thereby restoring redox balance and attenuating intestinal inflammation. Upward arrows indicate activation or increased expression, whereas downward arrows indicate inhibition or reduction. Abbreviations: ARE, Antioxidant Response Element; GST, Glutathione S-Transferase; HO-1, Heme Oxygenase-1; IκB, Inhibitor of κB; IKK, IκB Kinase; IL-1β, Interleukin-1β; IL-6, Interleukin-6; Keap1, Kelch-like ECH-associated Protein 1; NF-κB, Nuclear Factor Kappa B; NQO1, NAD(P)H Quinone Dehydrogenase 1; Nrf2, Nuclear Factor Erythroid 2-Related Factor 2; ROS, Reactive Oxygen Species; TNF-α, Tumor Necrosis Factor-α. Created in BioRender. Zeng, L. (2026) https://BioRender.com/sbjwr3e.

Oxidative stress and inflammation are closely interconnected. In the pathogenesis of IBD, the accelerated apoptosis of colonic mucosal epithelial cells and the delayed apoptosis of inflammatory cells simultaneously contribute to the persistent inflammation seen in IBD (Mansouri et al., 2025). The gastrointestinal tract is a major source of ROS production (Moura et al., 2015). Due to the low levels of antioxidants in the intestinal mucosa, when ROS are produced in excess, they attack intestinal epithelial cells, leading to membrane damage and functional disturbances. This can further result in tissue damage, generate inflammatory mediators via lipid peroxidation, and exacerbate the inflammatory response in IBD (Bhattacharyya et al., 2014; Chen and Zhong, 2014). Clinical studies have shown that mitochondrial oxidative stress in IBD patients leads to mitochondrial damage, reduced energy supply, and consequently, apoptosis. This results in impaired colonic mucosal barrier function, accelerated apoptosis of colonic epithelial cells, persistent tissue damage, and secondary malabsorption and electrolyte secretion dysfunction, thereby contributing to the progression of the disease (Chang, 2016). At the same time, lipid ROS and iron accumulation can drive ferroptosis, a form of programmed cell death primarily characterized by mitochondrial shrinkage and the reduction or disappearance of mitochondrial cristae (Dixon et al., 2012). The tight junctions of intestinal epithelial cells form the intestinal mucosal barrier. When ferroptosis occurs in intestinal epithelial cells, the barrier integrity is compromised, allowing large amounts of pro-inflammatory mediators to enter the intestine, stimulating the immune system and triggering inflammatory damage. Recent studies further demonstrated that mitochondrial damage-associated molecular patterns (mtDAMPs), particularly leaked mtDNA, can activate the cyclic GMP–AMP synthase-stimulator of interferon genes (cGAS-STING) pathway, thereby amplifying intestinal innate immune responses and type I interferon signaling in IBD. Excessive mitochondrial ROS production facilitates mtDNA oxidation and cytosolic release, which subsequently promotes STING-mediated inflammatory cascades and epithelial injury (West and Shadel, 2017). It has been confirmed that IBD patients exhibit mitochondrial dysfunction in epithelial cells, accompanied by increased mitochondrial-derived ROS production (Dashdorj et al., 2013; Wang et al., 2014). Moreover, studies have shown that in DSS-induced UC mice, Fe2+ accumulation, increased ROS production, and elevated levels of ferroptosis-related proteins occur (Wu Y. et al., 2023). In conclusion, mitochondrial oxidative stress plays a significant role in the pathogenesis of IBD (Figure 2).

### 3.3 IBD and mitochondrial quality control system dysfunction

Mitochondria play a crucial role in regulating energy metabolism and maintaining redox balance, while also being indispensable in vital cellular processes such as proliferation and differentiation. To uncover the specific molecular mechanisms underlying various cellular activities, a complex mitochondrial quality control system has gained increasing attention. This system includes key processes such as mitophagy, mitochondrial dynamics, and mitochondrial biogenesis (Liu X. et al., 2024). These processes play an essential role in the onset, progression, and outcomes of IBD.

#### 3.3.1 Abnormal mitophagy

Mitophagy is a self-digestion phenomenon widely present in eukaryotes and serves as a key mechanism for mitochondrial quality control and intracellular balance. As a negative regulatory mechanism, mitophagy removes aging, damaged, and dysfunctional mitochondria, preventing the release of harmful substances such as apoptotic proteins and ROS before mitochondrial apoptosis. This is crucial for maintaining mitochondrial homeostasis and meeting the cell’s energy demands (Mizushima and Komatsu, 2011). It is regulated by mitochondrial phosphatase and PINK1 as well as cytosolic E3 ubiquitin ligase Parkin (Pellegrino and Haynes, 2015). Under various stress factors, the balance within mitochondria is easily disrupted, leading to dysfunction in normal mitochondrial physiological functions and triggering various diseases (Suomalainen and Battersby, 2018). High levels of mitochondrial ROS and the oxidative damage of cellular DNA, proteins, and lipids are major molecular signals for mitophagy (Dashdorj et al., 2013). Damaged mitochondria are enveloped by autophagic vesicles with a double membrane, which then fuse with lysosomes for degradation, completing the process of mitophagy (Jin and Youle, 2012).

**FIGURE 3:**
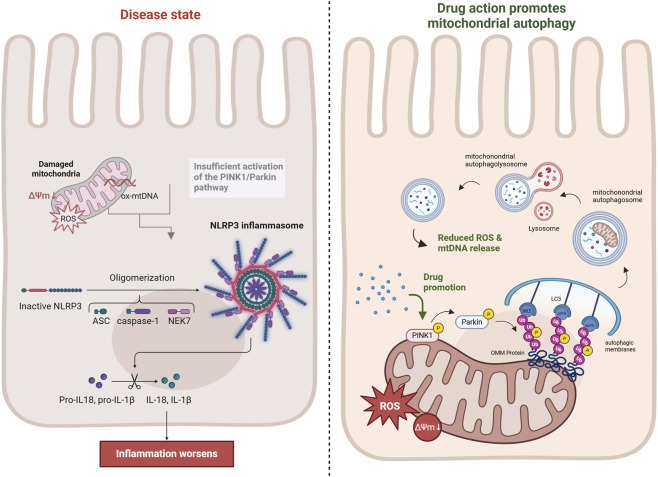
Regulating mitophagy, reducing ROS concentrations, inhibiting NLRP3 inflammasome activation, and maintaining immune cell homeostasis (Li et al., 2022; Lu et al., 2023; Picca et al., 2023) are now recognized as key strategies for preventing and treating IBD (Rossmann et al., 2021; Kong et al., 2024). The figure illustrates the proposed mechanism by which drug intervention alleviates intestinal inflammation through enhancement of mitophagy. In the disease state, damaged mitochondria release excessive ROS and oxidized mitochondrial DNA (ox-mtDNA), accompanied by insufficient activation of the PINK1/Parkin pathway, leading to NLRP3 inflammasome assembly and maturation of IL-1β and IL-18, thereby aggravating inflammation. Drug action promotes PINK1/Parkin-mediated mitophagy, facilitates autophagosome and autolysosome formation, and reduces ROS and mitochondrial DNA release, ultimately suppressing NLRP3 inflammasome activation and inflammatory responses. Abbreviations: ASC, Apoptosis-Associated Speck-like Protein Containing a CARD; IL-1β, Interleukin-1β; IL-18, Interleukin-18; LC3, Microtubule-Associated Protein 1 Light Chain 3; mtDNA, Mitochondrial DNA; NLRP3, NOD-like Receptor Family Pyrin Domain Containing 3; ox-mtDNA, Oxidized Mitochondrial DNA; PINK1, PTEN-Induced Kinase 1; ROS, Reactive Oxygen Species; ΔΨm, Mitochondrial Membrane Potential. Created in BioRender. Zeng, L. (2026) https://BioRender.com/3l48l6x.

Mitophagy is considered a protective mechanism against inflammatory damage in IBD. ROS and mtDNA released from damaged mitochondria can activate the intracellular NLRP3 inflammasome (López-Armada et al., 2013), while mitophagy can reduce ROS by removing dysfunctional mitochondria, thereby alleviating the onset and progression of inflammation (Cabrera et al., 2013). Studies have found that in IBD patients, the accumulation of damaged mitochondria in intestinal tissues increases, which correlates with the severity of the disease (Liang et al., 2022). The intestinal tissue requires large amounts of ATP, and mitochondria are highly developed in these cells. When these cells experience stress responses, mitophagy is induced to clear dysfunctional mitochondria, thus reducing the level of cellular stress (Wu and Chen, 2015). At the same time, mitophagy and its regulatory mechanisms are involved in maintaining intestinal homeostasis and repair. It contributes to the development and function of goblet cells, and defects in this process have a significant impact on Paneth cells, as well as on maintaining the intestinal epithelial barrier function (Khaloian, S. et al., 2020). Some studies have shown that mitochondrial damage and ROS production induce mitophagy, but in the intestinal tissues of IBD patients, mitophagy is significantly insufficient, leading to the failure to clear and repair damaged mitochondria in a timely manner. This further exacerbates mitochondrial dysfunction and oxidative stress, driving the progression of IBD. Other studies suggest that inducing various pathways to promote mitophagy can effectively prevent and alleviate IBD to some extent. Therefore, mitophagy is closely related to the onset and development of IBD. Dysregulation of mitophagy may lead to abnormal mitochondrial accumulation and increased inflammation, further promoting the progression of IBD (Figure 3).

#### 3.3.2 Abnormal mitochondrial fusion and fission functions

Mitochondria are constantly undergoing fusion and fission, a dynamic process known as mitochondrial dynamics. The dynamic balance between mitochondrial fusion and fission plays a crucial role in maintaining normal mitochondrial structure and function, as well as the homeostasis of the mitochondrial network (Eisner et al., 2018). Under physiological conditions, mitochondrial fusion facilitates the mixing of healthy and dysfunctional mitochondria, forming interconnected mitochondrial networks. This process mediates communication with the endoplasmic reticulum, participates in the dilution of mtDNA and oxidized proteins, and maintains cellular phosphorylation levels (Cao et al., 2021). On the other hand, mitochondrial fission separates dysfunctional mitochondria from the healthy mitochondrial pool, allowing for their removal through mitophagy and completing mitochondrial renewal. However, excessive mitochondrial fission can promote the generation of mitochondrial ROS, induce mitochondrial dysfunction, and subsequently trigger an inflammatory response (Pickles et al., 2018).

**FIGURE 4:**
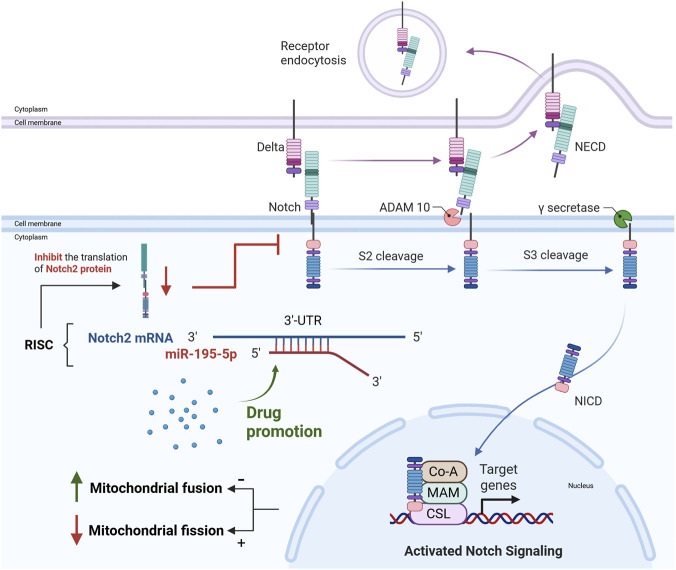
Mitochondrial dynamics imbalance is a core pathological process in IBD, and its intervention may become a therapeutic target for IBD (Zhang et al., 2021). The figure illustrates the proposed mechanism by which drug intervention modulates mitochondrial dynamics through the miR-195–5p/Notch pathway. Drug action promotes miR-195–5p expression, facilitating its binding to the 3′-UTR region of Notch2 mRNA and inhibiting Notch2 protein translation. Suppression of Notch signaling reduces receptor cleavage and downstream target gene transcription, thereby regulating mitochondrial homeostasis by enhancing mitochondrial fusion and reducing mitochondrial fission. Upward arrows indicate increased activity or expression, whereas downward arrows indicate inhibition or reduction. Abbreviations: 3′-UTR, 3′-Untranslated Region; ADAM10, A Disintegrin and Metalloproteinase Domain-Containing Protein 10; Co-A, Co-Activator; CSL, CBF1/Suppressor of Hairless/LAG-1; MAM, Mastermind-like Protein; miR-195–5p, MicroRNA-195–5p; NECD, Notch Extracellular Domain; NICD, Notch Intracellular Domain; RISC, RNA-Induced Silencing Complex. Created in BioRender. Zeng, L. (2026) https://BioRender.com/47wcaom.

Studies have shown that reduced mitochondrial fusion and excessive mitochondrial fission can stimulate the development of IBD by altering energy metabolism, oxidative stress, and cell death (Pickles et al., 2018). Mitochondrial dynamics imbalance, particularly excessive mitochondrial fission, can reduce energy production and increase oxidative stress, further diminishing cellular viability (Wang et al., 2022). In contrast, mitochondrial fusion helps mix mitochondrial contents (including DNA and respiratory chain complexes), participates in the repair of damaged mitochondria, reduces local oxidative stress levels, and further promotes mucosal repair (Lan et al., 2023). Mancini et al. reported that in mice with DSS-induced intestinal inflammation, the mRNA levels of Drp1 and Fis1 were elevated, triggering excessive mitochondrial fission (Mancini et al., 2020). Other studies have observed ZBP1-PANoptosis and Drp1-mediated mitochondrial fission in the intestinal epithelial cells of IBD patients, which are positively correlated with disease severity (Ye et al., 2024). Chojnacki et al. detected fragmented mitochondria in the colon tissue of DSS-induced colitis mice, and compared to wild-type mice and healthy controls, confirmed the correlation between mitochondrial fission and inflammation (Chojnacki et al., 2023). Moreover, drug regulation can improve impaired mitochondrial dynamics, thus demonstrating therapeutic potential for IBD (Taner et al., 2025). The mitochondrial fission inhibitor P110 significantly promoted mucosal healing in DSS-induced colitis mice, and the use of saquinavir inhibited mitochondrial fission, enhancing therapeutic effects in mice (Wang et al., 2021). Therefore, abnormal mitochondrial fusion and increased fission levels may drive the onset of IBD (Figure 4).

#### 3.3.3 Abnormal mitochondrial biogenesis

Mitochondrial biogenesis is the process through which new mitochondria are produced from existing ones through growth and fission, under the combined regulation of nuclear DNA and mtDNA. This process ensures mitochondrial renewal and provides cells with a sufficient pool of healthy mitochondria, playing a critical role in maintaining cellular metabolic homeostasis. The core of mitochondrial biogenesis involves the transcription and translation of mtDNA, expansion of mitochondrial membranes, and synthesis of oxidative phosphorylation-related proteins (Herrmann et al., 2012; Zhu et al., 2013). There are variations in mitochondrial quantity, size, and quality across different cells, and these changes reflect the current metabolic state of the cell (Kunz, 2003). Peroxisome proliferator-activated receptor gamma coactivator 1-alpha (PGC-1α) is the central regulator of mitochondrial biogenesis. It drives biosynthesis by activating various transcription factors such as nuclear respiratory factor-1 (NRF-1) and nuclear respiratory factor-2 (NRF-2). These factors not only control the expression of nuclear genes encoding mitochondrial proteins but also interact with mitochondrial transcription factor A (Tfam), promoting the transcription and replication of mtDNA, synthesizing the proteins required for mitochondrial function, and forming healthy mitochondria. PGC-1α is highly expressed in the intestinal epithelium (Jornayvaz and Shulman, 2010; Gorman et al., 2016).

**FIGURE 5:**
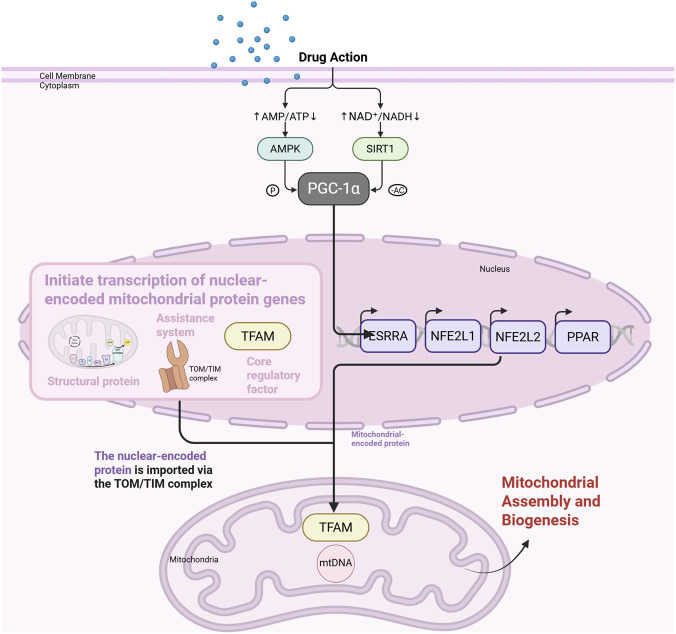
Regulating mitochondrial biogenesis effectively suppresses excessive inflammasome activation, reduces mitochondrial dysfunction, and alleviates intestinal mucosal damage (Nan et al., 2025). The figure illustrates the proposed mechanism by which drug intervention promotes mitochondrial biogenesis through upregulation of PGC-1α expression. Drug action activates AMPK and SIRT1 signaling in response to altered AMP/ATP and NAD+/NADH ratios, leading to phosphorylation and deacetylation of PGC-1α. Activated PGC-1α cooperates with transcription factors, including ESRRA, NFE2L1, NFE2L2, and PPAR, to initiate transcription of nuclear-encoded mitochondrial genes. TFAM-mediated mitochondrial DNA transcription and protein import through the TOM/TIM complex further contribute to mitochondrial assembly and biogenesis. Abbreviations: AMPK, AMP-Activated Protein Kinase; ESRRA, Estrogen-Related Receptor Alpha; mtDNA, Mitochondrial DNA; NFE2L1, Nuclear Factor, Erythroid 2 Like 1; NFE2L2, Nuclear Factor, Erythroid 2 Like 2; PGC-1α, Peroxisome Proliferator-Activated Receptor Gamma Coactivator-1 Alpha; PPAR, Peroxisome Proliferator-Activated Receptor; SIRT1, Sirtuin 1; TFAM, Mitochondrial Transcription Factor A; TOM/TIM complex, Translocase of the Outer/Inner Mitochondrial Membrane Complex. Created in BioRender. Zeng, L. (2026) https://BioRender.com/98s9bzj.

Studies have shown that linagliptin regulates mitochondrial biogenesis by promoting the AMPK/SIRT1/PGC-1α pathway, significantly reducing colonic inflammation (El-Ghannam et al., 2022). Other studies indicate that in IBD mice, the expression levels of PGC-1 and TFAM genes are significantly lower. However, in linoleic acid or anthocyanin treatment groups, the expression of PGC-1 and TFAM genes and proteins was significantly increased, promoting mitochondrial biogenesis and thereby contributing to therapeutic effects on IBD (Alattar et al., 2022; Alharbi et al., 2024). Clinical data have also shown that PGC-1α levels in surgical samples from colitis patients are lower than those in the control group (Cunningham et al., 2016). Therefore, mitochondrial renewal achieved through mitochondrial biogenesis may play an important role in the prognosis of IBD (Figure 5).

### 3.4 IBD and the gut microbiota-mitochondrial axis

The imbalance of the “gut microbiota-mitochondria axis” plays a critical role in the progression of IBD. In recent years, the crosstalk between intestinal epithelial cell mitochondria and the gut microbiota has gradually been revealed as a core regulatory point for intestinal homeostasis (Haque et al., 2024). This axis forms a complex bidirectional regulatory network: on one hand, the gut microbiota directly regulates mitochondrial function through its metabolic products; on the other hand, mitochondrial dysfunction can reshape the intestinal microenvironment. The disruption of the dynamic balance between these two elements can create a vicious cycle, driving the onset and progression of IBD.

The metabolic products of the gut microbiota are key signaling molecules in the regulation of mitochondrial function. Under physiological conditions, short-chain fatty acids (SCFAs) such as butyrate, acetate, and propionate, produced by the fermentation of dietary fiber by the gut microbiota, serve as the primary energy source for colonic epithelial cells (Mukhopadhya and Louis, 2025). Butyrate enters colonic epithelial cells through the monocarboxylate transporter (MCT1), where it is β-oxidized in the mitochondria to produce acetyl-CoA, which then enters the tricarboxylic acid cycle to drive oxidative phosphorylation and ATP production (Clausen and Mortensen, 1994). Additionally, butyrate can activate the AMPK-PGC1α-NRF1-TFAM axis, promoting mitochondrial biogenesis and restoring bioenergetic function, thereby enhancing mitochondrial respiration (Gonçalves and Martel, 2013; Li et al., 2022a). Several studies have shown that in IBD patients, the number of butyrate-producing bacteria, such as *Faecalibacterium prausnitzii* and *Roseburia* species, is significantly reduced (Sokol et al., 2009; Mirsepasi-Lauridsen et al., 2019). Dysbiosis leads to decreased SCFA production, and the downregulation of MCT1 reduces the absorption of butyrate by the mucosa, resulting in energy metabolism dysfunction in colonic epithelial cells and impaired barrier function (Thibault et al., 2010; Daisley et al., 2021).

At the same time, harmful metabolites produced by the overgrowth of opportunistic pathogens can directly damage mitochondrial function. Sulfate-reducing bacteria (SRB), such as *Desulfovibrio piger* and *Bilophila wadsworthia*, produce hydrogen sulfide (H_2_S), which inhibits the activity of mitochondrial complex IV, blocks the electron transport chain, and causes ATP synthesis dysfunction (Kushkevych et al., 2019; Kushkevych et al., 2020). *Bilophila wadsworthia*-mediated taurine metabolism produces H_2_S, which can damage mtDNA and impair mitophagy by inhibiting the ULK1 complex (Davies et al., 2024). Furthermore, infection with *Escherichia coli* LF82 in intestinal epithelial cells typically results in mitochondrial swelling, reduced mitochondrial membrane potential and ATP levels, and mitochondrial network fragmentation. Using mitochondrial fission inhibitors such as Mdivi1 or P110 can partially reduce mitochondrial fragmentation induced by *E. coli* LF82 in the short term (Mancini et al., 2021).

At the same time, mitochondrial dysfunction can have a reciprocal effect on the gut microbiota, reshaping the microbial community structure. Research has shown that in a mouse model with specific knockout of mitochondrial molecular chaperone Hsp60 in intestinal epithelial cells (Hsp60^Δ/ΔIEC^), there is an expansion of metabolic adaptive opportunistic pathogens, such as *Bacteroides*. However, this damage completely disappears in a germ-free environment, proving that mitochondrial dysfunction in intestinal epithelial cells exacerbates intestinal inflammation by remodeling the gut microbiota structure (Urbauer et al., 2024). Transcriptional analysis reveals that mitochondrial-damaged intestinal epithelial cells highly express oxidative stress-related genes (such as Ido1, Nos2, Duox2), and these gene signatures are also significantly enriched in the active inflammatory samples from CD patients. Mitochondrial-derived signaling molecules, such as ROS and mtDNA, can also modulate the intestinal immune microenvironment, indirectly influencing microbial colonization and composition (Gupta et al., 2026). When mitochondrial dysfunction occurs, excessive ROS production can activate inflammatory pathways like NF-κB, altering the intestinal mucosal microenvironment and creating favorable conditions for the colonization of pathogenic bacteria (Hirose et al., 2023).

## 4 Research progress on the treatment of IBD through the regulation of mitochondrial dysfunction by active metabolites of TCM

TCM formulations that tonify the spleen and augment qi, along with their active metabolites (such as Astragalus (Zheng et al., 2022)), can both promote mitochondrial biogenesis and mitigate oxidative damage. These formulas address the core issues of energy deficiency and oxidative damage in UC, simultaneously balancing “supporting the righteous” and “expelling the evil.” Heat-clearing, damp-dispelling, and detoxifying Chinese botanical drugs (such as Scutellaria baicalensis (Miao, 2021)) induce mitophagy, while spleen-tonifying and qi-boosting botanical drugs (such as Codonopsis pilosula (Fang et al., 2023)) regulate mitochondrial dynamics, exemplifying the TCM principle of “treating both the root and the branch.” With their multi-metabolite, multi-target, and holistic regulation advantages, TCM has shown significant potential in improving the pathological processes of IBD through mitochondrial function modulation.

The following review systematically discusses the latest research on the active metabolites of Chinese medicine, including alkaloids, terpenoids, saponins, quinones, flavonoids, polysaccharides, and polyphenols, and their mechanisms in regulating mitochondrial functions to treat IBD. This aims to provide a theoretical basis for the development of new Chinese medicine-based drugs targeting mitochondrial dysfunction in IBD.

It should be emphasized that many of the natural compounds discussed in this review, including resveratrol, curcumin, luteolin, genistein, and gallic acid, are not unique to TCM and occur widely across multiple plant taxa worldwide. These compounds are included because they are present in medicinal plants commonly used in TCM and have been investigated in TCM-related pharmacological studies. Therefore, the therapeutic relevance discussed herein should not be interpreted as evidence of chemical uniqueness to TCM.

Although the active metabolites discussed in this review are classified according to their chemical characteristics, increasing evidence suggests that their therapeutic effects converge on a limited number of mitochondrial regulatory mechanisms. To provide a more integrated mechanistic perspective, representative metabolites are categorized according to their primary mitochondrial targets and regulatory pathways in Table 1.

**TABLE 1:** Classification of active metabolites from TCM according to mitochondrial regulatory mechanisms in IBD.

Mitochondrial regulatory mechanism	Representative metabolites	Major molecular targets/Pathways
Energy metabolism restoration	Berberine, AT III, AS-IV, PNS, EP-1, DAPP	ATP production
Oxidative stress regulation	Berberine, Matrine, CEO, Ursolic acid, Andro, Celastrol, PNS, Rhein, Dan-shenone IIA, Baicalin, Luteolin, Hesperidin, Cardamom, Myricetin, Puerarin, EP-1, Gallic acid, CGA, Resveratrol, Geniposide	ROS, Nrf2/HO-1, SOD
Mitophagy regulation	Evodiamine, Andro, Celastrol, PNS, Ginsenoside Rb, GLP, APS, Resveratrol, Curcumin, Coixol, Myricanol, Bergapten	PINK1/Parkin, LC3
Mitochondrial dynamics regulation	AT I, Luteolin	Drp1, Mfn1, Mfn2
Mitochondrial biogenesis	DMB, AT III, Genkwanin, DAPP, Genistein, Sulforaphane	PGC-1α, NRF1, TFAM
Gut microbiota–mitochondria axis	DHE, Paeoniflorin, PNS, FLP, Sauchinone	SCFAs, microbial metabolites

### 4.1 Alkaloids

Alkaloids are a class of nitrogen-containing organic metabolites found in various organisms, possessing diverse biological activities. They are typically classified into subgroups such as isoquinoline alkaloids, indole alkaloids, and pyridine alkaloids (Peng et al., 2019).

Berberine (BBR) is an isoquinoline alkaloid derived from plants such as *Coptis chinensis* Franch. And *Phellodendron amurense*, and is commonly used in TCM to treat diarrhea (Lu et al., 2021). Several studies have shown that BBR can alleviate IBD by regulating mitochondrial membrane damage, exerting anti-inflammatory effects, reducing oxidative stress, and activating autophagy (Zhang et al., 2017; Cao et al., 2022; Xu et al., 2022; Yu et al., 2024) (Table 2).

**TABLE 2:** Mechanism and efficacy of BBR in regulating mitochondrial dysfunction for the prevention and treatment of IBD.

Active metabolites in TCM	Mechanism of action	Mitochondrial regulatory dimensions	Effectiveness	Literature cited
Berberine (BBR)	Inhibition of the PLA2-COX-2-PGE2-EP2 pathway in UC via the gut microbiota	Energy Metabolism and Oxidative Stress	PLA2 is closely associated with mitochondrial membrane damage and the initiation of oxidative stress	Yu et al. (2024)
	Significantly suppressed the expression of IL-1β, IL-6, and tumor necrosis factor-α (TNF-α), reduced peroxidase levels in colonic samples, and increased levels of superoxide dismutase and catalase	Oxidative Stress	Anti-inflammatory and antioxidant stress	Zhang et al. (2017)
	Activate mitophagy through the AMPK/mTOR/ULK1 pathway	Mitophagy	Control inflammatory responses and alleviate colitis	Xu et al. (2022)

Matrine (MT) and oxidized matrine (oxymatrine, OMT) can alleviate IBD by modulating oxidative stress and mitigating ferroptosis. MT, a natural quinolizidine alkaloid, is the main active metabolite of *Sophora flavescens* Aiton and other leguminous plants, with pharmacological activities such as anticancer, antiviral, anti-inflammatory, and antioxidant properties (Zhang, 2019). DSS-induced colitis leads to oxidative stress-related intestinal injury and decreased antioxidant enzyme levels. However, oral MT treatment in DSS-induced colitis mice restores T-AOC, SOD, CAT, and MDA levels in the colon, suggesting that MT alleviates IBD by modulating intestinal oxidative stress levels (Mao et al., 2024). OMT, another alkaloid extracted from the root of *Sophora flavescens* Aiton, is similar to MT and is used to treat various acute or chronic inflammations (Zhou et al., 2014). OMT alleviates IBD by inhibiting inflammatory factors (IL-1β, IL-6, TNF-α, HMGB1, and NLRP3) and reducing iron deposition (Fe2+, GSH), thereby improving inflammation and ferroptosis (Gao et al., 2024).

Evodiamine, an indole alkaloid from *Evodia rutaecarpa* (Juss.) Benth., has therapeutic effects on colitis (Shi et al., 2018). Research has found that it can activate autophagy, attenuate inflammatory signaling, and inhibit the NF-κB pathway, thereby exerting protective effects in IBD (Shen P. et al., 2019; Ding et al., 2020). Dehydroevodiamine (DHE) can modulate gut microbiota structure. It reduces the abundance of opportunistic pathogens such as *Allobaculum*, *Clostridium*, and *Escherichia*, while increasing the abundance of beneficial bacteria such as *Lactobacillus* and *Bifidobacterium*. DHE exerts its anti-UC effects by targeting AKT1 and inhibiting the inflammatory and oxidative stress-related signaling pathways (Ma et al., 2024).

The root extract of *Coptis chinensis* Franch., de-methoxyberberine (DMB), is a protoberberine alkaloid that can inhibit TLR4-mitochondrial signaling and the maturation of IL-1β, reducing mitochondrial biogenesis and improving the inflammatory response (Zhao et al., 2022).

### 4.2 Terpenes

Terpenoid (also known as isoprenoids or terpenes) are a large class of small molecule secondary metabolites with isoprene as the basic structural unit, composed of repeating five-carbon isoprene units. According to the number of isoprene units, terpenoids in TCM can be roughly divided into sesquiterpenes, monoterpenes, diterpenes, and triterpenes (Yang et al., 2016). These compounds exhibit a wide range of biological activities, including antibacterial, antioxidant, antitumor, hepatoprotective, and anti-inflammatory effects (Germoush et al., 2020).

Atractyloside (AT), a sesquiterpene lactone in the terpenoid family, has been shown to play an active role in the prevention and treatment of IBD (Qin et al., 2021; Han et al., 2022). However, the mechanisms are different for the two forms of atractylenolide I (AT I) and atractylenolide III (AT III). AT I primarily exerts its effects by inhibiting Drp1-mediated mitochondrial fission and reducing mitochondrial damage through anti-inflammatory actions, while AT III alleviates IBD by modulating mitochondrial function in the colons of IBD mice, exerting anti-inflammatory and antioxidant effects, and repairing damaged mitochondria to restore intestinal epithelial barrier integrity (Table 3).

**TABLE 3:** Mechanism and efficacy of at regulation in preventing and treating mitochondrial dysfunction in IBD.

Active metabolites in TCM	Mechanism of action	Mitochondrial regulatory dimensions	Effectiveness	Literature cited
Atractylenolide I (AT I)	Inhibits Drp1-mediated mitochondrial fission and suppresses NLRP3 inflammasome activation	Mitochondrial dynamics	Reversing NLRP3-induced mitochondrial damage to maintain intestinal homeostasis	Qin et al. (2021)
Atractylenolide III (AT III)	Significantly increased the number of mtDNA copies in the colon of IBD mice, as well as the activity of Complex I and Complex IV.	Energy Metabolism and Oxidative Stress	Inhibit inflammation and oxidative stress	Han et al. (2022)
	Enhance PGC-1α expression to repair damaged mitochondria	Mitochondrial Biogenesis	Alleviate mitochondrial dysfunction and mitigate damage to the intestinal epithelial barrier	Han et al. (2022)

Additionally, various other terpenoid can regulate oxidative stress through anti-inflammatory effects and induction of mitophagy, thereby alleviating intestinal inflammation. Ethanol extract of *Chimonanthus nitens* Oliv (CEO), a traditional medicinal plant extract, has demonstrated anti-inflammatory and antioxidant effects in experimental colitis models. In rat models, CEO reduces IL-1β secretion, inhibits caspase-1 and restores ZO-1 expression, and NF-κB p65 phosphorylation, while decreasing levels of malondialdehyde (MDA) and enhancing the activity of superoxide dismutase (SOD) in intestinal tissues (Wan F. et al., 2021). Ursolic acid (UA), a pentacyclic triterpene extracted from rosemary and fruit peels, possesses anti-inflammatory, antioxidant, and anti-ulcer properties (Seo et al., 2018). It upregulates the expression of CAT and T-SOD, reducing ROS production in cells and alleviating mesangial cell injury (Wei et al., 2022). Andrographolide (Andro), the main active metabolite of *Andrographis paniculata* (Burm.f.) Nees, has significant anti-inflammatory effects (Yang et al., 2016). Andro suppresses oxidative stress and inflammation through the Nrf2/HO-1 pathway (Shu et al., 2024). It also induces mitophagy by inhibiting the PIK3CA-AKT1-mTOR-RPS6KB1 pathway, selectively clearing damaged mitochondria in cells to alleviate oxidative stress and inflammation (Li et al., 2022b). Celastrol, a natural pentacyclic triterpene found in *Tripterygium wilfordii* Hook. f., has neuroprotective and anti-inflammatory properties (Safwat El-Deeb et al., 2022). Celastrol controls DSS-induced colonic oxidative stress by reducing lipid peroxidation products (malondialdehyde and 4-hydroxy-2-nonenal) and enhancing antioxidant levels (reduced glutathione, glutathione-S-transferase, and superoxide dismutase) (Shaker et al., 2014). Celastrol also improves experimental colitis in IL-10 knockout mice by upregulating mitophagy through inhibition of the PI3K/Akt/mTOR signaling pathway (Zhao et al., 2015).

Paeoniflorin, a monoterpene glycoside metabolite extracted from the plant *Paeonia lactiflora* Pall., is widely recognized for its immune-regulating and anti-inflammatory effects in clinical settings (Zhang and Wei, 2020). It reduces the levels of indole-3-lactic acid (ILA) in the colon and alleviates colitis via the gut microbiota-ILA-epithelial autophagy axis (Fan et al., 2020).

### 4.3 Saponins

Saponins are natural surface-active glycosides composed of triterpene or steroidal structures, and are major metabolites in common botanical drug plants such as *Panax ginseng*, *Astragalus membranaceus*, and *Panax notoginseng*. They exhibit anti-inflammatory effects and play a role in regulating immune homeostasis (Dong et al., 2019). Astragaloside IV (AS-IV) and Panax notoginseng saponins (PNS) have been shown to alleviate IBD by regulating mitochondrial energy metabolism.

AS-IV, a natural saponin extracted from the leguminous plant *Astragalus membranaceus*, has antioxidant and immune-modulating effects (Li et al., 2017). It can alleviate the decrease in ATP levels and ATP synthase β subunit expression in colon tissues induced by TNBS (2,4,6-trinitrobenzene sulfonic acid) treatment, regulate energy metabolism, and promote mucosal healing in TNBS-induced colitis (Jiang et al., 2017).

PNS, an active metabolite extracted from the dried roots and rhizomes of *Panax notoginseng*, has multifaceted effects in alleviating intestinal inflammation (Dong et al., 2019). PNS restores mitochondrial membrane potential, increases LC3II/I expression, reduces p62 and OPTN levels, and significantly increases Parkin levels, enhancing autophagic flux (Jiang et al., 2022). Additionally, PNS reduces malondialdehyde (MDA) and myeloperoxidase (MPO) activity, while increasing the expression of catalase (CAT) and superoxide dismutase (SOD) in intestinal tissues, thus alleviating oxidative stress-induced damage (Luo et al., 2021). PNS also increases the Firmicutes/Bacteroidetes (F/B) ratio, enriches beneficial bacteria such as *Bifidobacterium* and *Lactobacillus*, inhibits the growth of pathogenic bacteria like *Verrucomicrobiota*, and significantly reduces levels of characteristic metabolites such as lysophosphatidylinositol (LysoPI). These effects help regulate the gut microbiota composition and maintain intestinal barrier function (Song et al., 2025).

Ginsenoside Rb, a triterpene saponin extracted from the roots, stems, leaves, and fruits of *Panax* species, is classified into three types based on its structural features: protopanaxadiol (PPD), protopanaxatriol (PPT), and oleanolic acid (OA) (Baatar et al., 2018). Ginsenoside Rb exerts its antioxidant effects by targeting core regulatory factors in the nuclear factor kappa-light-chain-enhancer of activated B cells (NF-κB) signaling pathway, alleviating apoptosis and mitochondrial dysfunction induced by oxidative stress in cells (Dong et al., 2022). Furthermore, it activates the AMP-activated protein kinase (AMPK)/ULK1/p62 axis, promoting mitophagy, and induces the deactivation of the NLRP3 inflammasome. This reduces the secretion of IL-1β by macrophages and suppresses colitis in a DSS-induced mouse model (Liu et al., 2018).

### 4.4 Quinones

Quinones are a class of natural metabolites with unsaturated cyclic diketone structures, mainly categorized into four types: *benzoquinone*, *naphthoquinone*, *anthraquinone*, and *phenanthraquinone*. They possess various pharmacological activities, including antibacterial, anti-inflammatory, and antiviral effects (Yusuf et al., 2019).

Rhein is an anthraquinone metabolite extracted from the medicinal botanical drug *Rheum palmatum* L (commonly known as rhubarb), and it is widely found in various TCM. Rhein has notable anti-inflammatory properties (Wu et al., 2017). Rhein exerts anti-inflammatory and redox-regulatory effects that contribute to the attenuation of intestinal inflammation in experimental colitis models (Zhou et al., 2022).

Dan-shenone IIA is a phenanthraquinone metabolite extracted from the *Salvia miltiorrhiza* Bunge (Danshen), a plant of the Lamiaceae family (Guo et al., 2020). Dan-shenone IIA ameliorates inflammatory injury and oxidative imbalance in TNBS-induced colitis models through multi-target mitochondrial protective mechanisms (Zhang et al., 2015).

### 4.5 Flavonoids

Flavonoids are widely found in natural plants and possess various biological activities, including antioxidant, anti-inflammatory, and antiviral effects. Many flavonoid can repair the integrity of the intestinal mucosal barrier by inhibiting the activation of inflammatory signaling pathways such as NF-κB, and by increasing the expression of antioxidant enzymes through Nrf2-mediated antioxidant stress pathways. This reduces oxidative stress damage and the production of inflammatory mediators, as seen with metabolites such as *baicalin* (Shen J. et al., 2019), *luteolin* (Nunes et al., 2017), *hesperidin* (Guo et al., 2019), *cardamomin* (Ali et al., 2017), *myricetin* (Cho et al., 2016), and *Puerarin* (Jeon et al., 2020) (Table 4).

**TABLE 4:** Mechanism and efficacy of flavonoids in regulating mitochondrial oxidative stress for the prevention and treatment of IBD.

Active metabolites in TCM	Mechanism of action	Effectiveness	Literature cited
Baicalin	Inhibits NF-κB and PI3K/Akt signaling pathways, enhances CAT, SOD, and GSH-Px activity in inflamed colonic tissue, and suppresses TNBS-induced increases in ROS and malondialdehyde levels	Control inflammatory responses and mitigate oxidative stress damage	Shen J. et al. (2019)
Luteolin	Blocking NF-κB pathway transduction activates the Nrf2 signaling pathway, increasing the expression of HO-1, NQO1, SOD, and CAT in colonic tissue while downregulating malondialdehyde levels	Enhance the body’s ability to resist oxidative stress	Nunes et al. (2017)
Hesperidin	Upregulate the Nrf2 antioxidant pathway to increase the protein expression of HO-1 and NQO1	Enhancing antioxidant pathways to protect against intestinal inflammation	Guo et al. (2019)
Cardamom	Reduces levels of caspase-3, MPO, iNOS, COX-2, and malondialdehyde; inhibits TNF-induced apoptosis and reduces oxidative stress	Regulate oxidative stress and inhibit apoptosis	Ali et al. (2017)
Myricetin	Inhibits NF-κB and STAT1 activation as well as Nrf2-mediated HO-1 expression, reduces nitric oxide production, decreases MPO and malondialdehyde levels, and increases SOD and GSH-Px expression	Inhibits the production of pro-inflammatory mediators and exerts antioxidant effects	Cho et al. (2016)
Puerarin	Regulation of the Nrf2 pathway and antioxidant enzyme expression	Antioxidant effect	Jeon et al. (2020)

Among them, *luteolin* can improve mitochondrial dynamics by inhibiting the miR-195–5p/Notch signaling pathway, upregulating mitochondrial fusion factors (MFN1, MFN2), and inhibiting fission factors (Fis1, Crmp1). This supports energy metabolism needed for normal colonic tissue function and alleviates DSS-induced intestinal mucosal damage (Liu M. et al., 2025).

Genkwanin, another flavonoid, can upregulate the expression of SIRT1. Activated SIRT1 deacetylates and activates PGC-1α, thereby regulating mitochondrial biogenesis. Additionally, it inhibits ROS production, thereby alleviating oxidative stress, reducing pro-inflammatory cytokine production, and improving mitochondrial function in human intestinal epithelial cells (Chen et al., 2022).

### 4.6 Polysaccharides

Polysaccharides are known for their high efficacy, low toxicity, and broad biological activities. They are commonly used as active metabolites in the treatment of inflammatory bowel disease (IBD), exerting anti-inflammatory effects, modulating gut microbiota, and improving intestinal barrier function, among other mechanisms to alleviate intestinal inflammation (Zeng et al., 2019).

For example:

Flos Lonicera polysaccharide (FLP) can regulate the gut microbiota structure in IBD mouse models, increasing the abundance of beneficial bacteria. It also significantly affects the levels of mitochondrial function-related coenzyme Q10 (ubiquinone), catalyzing oxidative phosphorylation, thereby inhibiting oxidative stress and NF-κB pathway activation (Xia et al., 2025).

Hericium erinaceus polysaccharide (EP-1), extracted from the mycelium of *Hericium erinaceus*, reduces ROS levels and oxidative damage by increasing SOD enzyme activity, thereby enhancing oxygen consumption and ATP production. This increased respiratory activity accelerates the elimination of excess ROS substrates and promotes mitochondrial energy production, reducing epithelial cell apoptosis and strengthening the intestinal barrier, thus alleviating IBD (Wang et al., 2019).

Ganoderma lucidum polysaccharides (GLP) promote mitophagy by regulating the MAPK and AMPK/Akt/mTOR signaling pathways, and can also restore mitochondrial function (Liang et al., 2019).

Astragalus polysaccharide (APS), extracted from *Astragalus membranaceus*, plays a crucial role in anti-inflammatory, antioxidant, antiviral, and immune modulation. APS enhances the expression of AhR, and the activated AhR binds to the promoter of Becn1 to initiate the transcription of genes related to anti-inflammation and intestinal barrier repair, while promoting targeted autophagy, thus providing protective effects in colitis mouse models (Ying et al., 2024).

### 4.7 Polyphenols

Polyphenolic are primarily classified into flavonoids, phenolic acids, tannins, lignans, and stilbenes, and are known for their antioxidant, immune-modulating, and anticancer activities (Pandey and Rizvi, 2009; Zhou et al., 2016). Here are a few examples of how polyphenols interact with mitochondrial function in the context of IBD:

Polyphenol-rich extracts derived from apple peels (DAPP) have shown strong potential in regulating mitochondrial energy metabolism and oxidative stress. Studies (Yeganeh et al., 2018) have found that DAPP can improve mitochondrial ultrastructure, enhance the activity of mitochondrial respiratory chain complexes I and IV, and upregulate key mitochondrial biogenesis regulators such as PGC-1α, thereby alleviating mitochondrial dysfunction.

Gallic acid (GA), a phenolic acid metabolite, has anti-inflammatory and antioxidant properties. It can reduce ROS levels induced by lipopolysaccharide (LPS) in Caco-2 cells, restoring the activity of antioxidant enzymes like SOD and CAT, as well as glutathione levels (Chu et al., 2024).

Chlorogenic acid (CGA), a phenolic acid found in various plants and fruits, can increase the expression of antioxidant enzymes such as SOD and CAT, while decreasing malondialdehyde (MDA) levels and ROS generation, thus reducing oxidative stress damage (Wan M. et al., 2021).

Resveratrol (RSV), a natural polyphenol found in grapes, knotweed, and berries, has significant therapeutic potential. RSV mitigates oxidative stress-induced gut barrier damage through the PI3K/Akt-mediated Nrf2 signaling pathway (Zhuang et al., 2019). It also inhibits ferroptosis via the Nrf2/Slc7a11/Gpx4 pathway, preventing DSS-induced colitis (Zheng et al., 2024). Additionally, RSV upregulates PINK1, Parkin, and LC3-II/LC3-I, thereby activating mitophagy (Cao et al., 2019).

Curcumin, derived from turmeric, is another polyphenolic metabolite that promotes Parkin-dependent mitophagy via the AMPK-TFEB signaling pathway. It helps alleviate gut barrier and mitochondrial damage caused by oxidative stress (Cao et al., 2020).

Coixol, found in Coix, promotes mitophagy and inhibits the IL17A signaling pathway, preventing damaged mitochondria from accumulating and triggering secondary inflammatory responses, thereby achieving therapeutic effects for IBD (Chen et al., 2025).

Sauchinone, a lignan obtained from *Saururus chinensis*, regulates mitochondrial function by upregulating NAD(P)H dehydrogenase quinone 1 (NQO1) expression in DSS-induced UC mice. It inhibits NF-κB activation and reduces inflammation in colon tissues, protecting the gut mucosal barrier. Additionally, sauchinone stimulates the growth of Firmicutes and inhibits the proliferation of Proteobacteria and Bacteroidetes, helping restore gut microbiota diversity and balance (Wu et al., 2022).

### 4.8 Other active metabolites in TCM

Geniposide, iridoid glycoside extracted from Gardenia jasminoides, can inhibit the phosphorylation of IκB-α and p65, as well as the degradation of IκB-α. Additionally, it enhances the phosphorylation and transcriptional activity of Nrf2 in LPS-treated Caco-2 cells. It also interacts with KEAP1, disrupting the KEAP1-Nrf2 interaction, preventing Nrf2 degradation, and activating the Nrf2/ARE signaling pathway. Ultimately, this results in the suppression of inflammation induced by redox imbalance, demonstrating significant anti-inflammatory and antioxidant activities (Zhuge et al., 2023).

Myricanol (MYR), a medium-chain alcohol metabolite, has been shown to alleviate LPS-induced mitochondrial damage in cells through the ANXA1/PINK1/Parkin pathway. It also reduces the generation of mitochondrial reactive oxygen species (mtROS), decreases cell death, and mitigates structural damage in intestinal epithelial cell (Li et al., 2025).

Bergapten (BP), a naturally occurring coumarin metabolite, promotes mitophagy and maintains mitochondrial homeostasis. It suppresses inflammatory responses and pyroptosis, thereby exhibiting therapeutic effects in a mouse model of colitis (Li et al., 2025).

Genistein and Sulforaphane can significantly upregulate the expression and protein levels of PGC-1α and mitochondrial transcription factor A (TFAM), thereby regulating energy metabolism and mitochondrial biogenesis, which contributes to their therapeutic effects on IBD (Alattar et al., 2022; Alharbi et al., 2024).

## 5 Clinical evidence of active metabolites from TCM in IBD

Several active metabolites from TCM have demonstrated preliminary clinical benefits in patients with IBD, particularly UC, suggesting potential translational relevance beyond preclinical models. However, most currently available clinical studies mainly evaluated symptomatic improvement and inflammatory indices, while direct assessment of mitochondrial function-related biomarkers in human tissues remains largely lacking.

Among these compounds, Curcumin has shown the most substantial clinical evidence. In a randomized, double-blind, placebo-controlled multicenter trial, curcumin combined with mesalamine significantly reduced relapse rates and improved clinical and endoscopic indices in patients with quiescent UC (Hanai et al., 2006). Additional randomized controlled trials further demonstrated that curcumin supplementation could improve clinical remission and mucosal healing in patients with mild-to-moderate active UC (Lang et al., 2015). More recently, a curcumin-QingDai combination also exhibited therapeutic efficacy in active UC patients in a placebo-controlled clinical trial (Ben-Horin et al., 2024). Nevertheless, these studies primarily focused on inflammatory outcomes and did not directly evaluate mitochondrial bioenergetics, oxidative phosphorylation-related parameters, or oxidative stress.

Resveratrol has also shown promising clinical potential in UC. In randomized placebo-controlled pilot studies, resveratrol supplementation significantly reduced disease activity and oxidative stress markers while improving quality of life in patients with active UC (Samsami-Kor et al., 2015; Samsamikor et al., 2016). Serum antioxidant capacity and superoxide dismutase activity were increased, whereas malondialdehyde levels were reduced following resveratrol administration. These findings indirectly support the possibility that mitochondrial redox homeostasis may participate in the therapeutic effects of resveratrol in human IBD. However, mechanistic validation in patient-derived tissues remains insufficient.

In addition, increasing evidence suggests that dietary polyphenol-based interventions may exert adjunctive therapeutic effects in UC patients. A recent multicenter randomized clinical trial demonstrated that Mediterranean diet combined with curcumin or resveratrol supplementation improved inflammatory status and quality of life in patients with mild-to-moderate active UC (Erol Doğan et al., 2024). However, whether these clinical benefits are mechanistically associated with mitochondrial quality control, mitochondrial biogenesis, or mitophagy remains unclear.

Despite these encouraging findings, the current clinical evidence remains insufficient to establish definitive efficacy. Most studies enrolled relatively small patient populations and were conducted over short treatment durations. Furthermore, substantial heterogeneity exists in formulations, dosages, concomitant medications, and outcome measures. Importantly, none of the currently available clinical trials directly evaluated mitochondrial function-related biomarkers, such as ATP production, respiratory chain activity, mitochondrial DNA integrity, or mitophagy markers. Therefore, the proposed mitochondrial mechanisms remain largely extrapolated from preclinical studies and require validation in well-designed translational clinical investigations.

## 6 Potential PAINS liabilities of active metabolites from TCM

While numerous active metabolites derived from TCM have demonstrated promising protective effects against mitochondrial dysfunction in experimental models of IBD, the interpretation of these findings requires careful consideration of potential pan-assay interference compound (PAINS) behavior. PAINS are molecules that may generate apparent biological activities through nonspecific mechanisms, thereby producing misleading results in biochemical and cell-based assays (Baell and Holloway, 2010; Aldrich et al., 2017).

Several metabolites discussed in this review belong to structural classes that have been frequently associated with PAINS-related liabilities. In particular, curcumin, luteolin, myricetin, gallic acid, genistein, and resveratrol warrant particular attention because their reported activities may arise partly from assay interference rather than target-specific mitochondrial modulation (Table 5). Curcumin possesses α,β-unsaturated carbonyl groups that may act as Michael acceptors and react nonspecifically with proteins. Luteolin and myricetin contain catechol-like structures capable of redox cycling, metal chelation, and assay interference. Gallic acid exhibits strong nonspecific antioxidant activity and metal-binding properties. Genistein is known to interact with multiple kinase families and may produce apparent target selectivity. Resveratrol has been reported to exhibit aggregation-dependent effects and broad target promiscuity. (Baell and Holloway, 2010; Capuzzi et al., 2017; Bolz et al., 2021).

**TABLE 5:** Potential PAINS-related liabilities of representative metabolites discussed in this review.

Metabolite	Structural feature associated with PAINS	Potential concern	Main evidence level	Validation priority
Curcumin	α, β-unsaturated carbonyl; polyphenol	Michael acceptor reactivity, fluorescence interference, and chemical instability	Cell, animal, clinical	High
Resveratro	Polyphenolic scaffold	Potential redox-related assay interference and target promiscuity	Cell, animal, clinical	High
Luteolin	Catechol flavonoid	Catechol-mediated redox cycling, metal chelation, and nonspecific interactions	Cell, animal	High
Myricetin	Polyhydroxylated flavonoid	Redox cycling, metal chelation, and aggregation tendency	Cell, animal	Moderate
Gallic acid	Phenolic acid	Antioxidant-dependent assay interference	Cell	Moderate
Genistein	Polyphenolic isoflavone	Potential nonspecific target modulation and pathway promiscuity	Cell, animal	Moderate
Rhein	Anthraquinone derivative	Quinone-associated redox activity and nonspecific oxidative stress modulation	Cell, animal	Moderate

PAINS-related liabilities were summarized according to published analyses of pan-assay interference compounds and assay artifacts associated with natural products (Baell and Holloway, 2010; Aldrich et al., 2017; Capuzzi et al., 2017; Bolz et al., 2021). The presence of these structural features does not invalidate the reported biological activities but highlights the need for rigorous target validation and orthogonal experimental approaches.

Since many mechanistic studies included in this review rely primarily on ROS measurements, mitochondrial membrane potential assays, Western blot analyses of signaling proteins, or antioxidant activity assessments, the possibility of assay interference should be carefully considered when interpreting causal relationships between specific metabolites and mitochondrial targets. Therefore, some reported mitochondrial protective mechanisms may require further validation using complementary genetic, pharmacological, and target-engagement approaches. Importantly, the presence of PAINS-associated structural alerts does not necessarily invalidate the reported biological activities of these compounds. Rather, it highlights the need for rigorous pharmacological validation and careful interpretation of experimental findings (Capuzzi et al., 2017).

Therefore, future studies should incorporate orthogonal assay systems, target-engagement validation, mitochondrial localization analyses, pharmacokinetic characterization, and human tissue-based verification strategies to distinguish genuine mitochondrial modulators from assay-dependent artifacts. Such approaches will be essential for improving the translational reliability of active metabolites from TCM targeting mitochondrial dysfunction in IBD.

## 7 Discussion

In summary, mitochondrial energy metabolism dysfunction, oxidative stress, abnormalities in mitochondrial quality control systems, and the imbalance of the “gut microbiota-mitochondria axis” play a crucial role in the pathogenesis and progression of IBD, making them potential therapeutic targets. Currently, glucocorticoids, amino-salicylate preparations, and immunosuppressive agents (such as thiopurines, methotrexate, and 6-mercaptopurine) are the most commonly used drugs for treating IBD, with their core mechanism mainly focused on inhibiting excessive immune inflammation to promote mucosal healing (Wangchuk et al., 2024). However, traditional treatments face limitations in promoting full recovery of cellular function, leading to challenges such as high relapse rates in IBD treatment. In contrast, Chinese medicine interventions present an alternative therapeutic approach. Various active metabolites of TCM have been shown protective effects in preclinical models by regulating mitochondrial dysfunction (in processes such as energy metabolism, oxidative stress, mitophagy, dynamics, and biogenesis), or by reshaping gut microbiota structure and optimizing microbial metabolic product profiles. Among these pathways, oxidative stress regulation appears to be the most extensively investigated mechanism. Numerous metabolites have been shown to reduce excessive mitochondrial ROS accumulation and improve antioxidant defenses. In contrast, studies focusing on mitochondrial dynamics and biogenesis remain relatively limited, suggesting that these areas warrant further investigation. Importantly, increasing evidence indicates that multiple mitochondrial pathways are interconnected and may collectively contribute to disease progression and therapeutic responses in IBD. Therefore, despite their structural diversity, active metabolites of TCM may exert protective effects through a shared network of mitochondrial regulatory mechanisms rather than through isolated signaling pathways. This mechanistic convergence highlights mitochondrial homeostasis as a promising therapeutic target for future IBD interventions.

Nevertheless, it should be emphasized that IBD is a highly heterogeneous and multifactorial disease, and mitochondrial dysfunction should not be considered the sole pathogenic driver. Current evidence suggests that genetic susceptibility, intestinal immune dysregulation, epithelial barrier impairment, environmental exposure, endoplasmic reticulum stress, and gut microbiota dysbiosis collectively contribute to disease initiation and progression. Mitochondrial dysfunction likely interacts dynamically with these pathogenic factors through complex bidirectional regulatory networks rather than functioning as an isolated mechanism. Therefore, future studies should integrate mitochondrial biology into broader multi-omics and systems-level frameworks to better elucidate the complex pathophysiological landscape of IBD.

Despite the promising preclinical findings, several important limitations currently hinder the translational application of active metabolites of TCM targeting mitochondrial dysfunction in IBD. First, most available studies rely heavily on chemically induced murine colitis models or immortalized intestinal epithelial cell lines, which incompletely recapitulate the complex immune heterogeneity, chronic relapsing nature, and patient-specific metabolic characteristics of human IBD. Second, many studies primarily assess indirect oxidative stress markers or inflammatory cytokines without rigorously validating mitochondrial-specific mechanisms, making it difficult to determine whether the observed therapeutic effects truly originate from direct modulation of mitochondrial function.

In addition, a considerable proportion of natural compounds discussed in current studies, particularly polyphenols, flavonoids, and quinone-related molecules, may exhibit PAINS-associated properties. Their reported biological activities could partially arise from nonspecific antioxidant effects, fluorescence interference, redox cycling, or promiscuous molecular interactions rather than target-specific pharmacological mechanisms. Moreover, poor bioavailability, rapid metabolism, and insufficient tissue-specific pharmacokinetic evaluation further limit the clinical translation potential of many active metabolites of TCM.

Future investigations should prioritize several key areas. First, studies should move beyond descriptive observations and identify direct mitochondrial targets of TCM-derived compounds, including PINK1, Parkin, DRP1, TFAM, and PGC-1α, rather than relying solely on downstream biomarkers. Second, clinically relevant validation systems, including patient-derived intestinal organoids, *ex vivo* intestinal tissues, and human biopsy-based mitochondrial profiling approaches, should be incorporated to improve translational relevance and bridge the gap between preclinical findings and human disease. Third, mitochondrial-specific functional biomarkers, such as mitochondrial respiratory complex activity, ATP production, mitochondrial membrane potential, OCR, mtDNA integrity, circulating mtDNA, and mitophagy flux, should be systematically integrated into clinical and translational studies to accurately evaluate mitochondrial-targeted therapeutic effects. Furthermore, future pharmacological investigations should emphasize target-engagement validation, mitochondrial localization analysis, and comprehensive pharmacokinetic and bioavailability characterization, as many natural compounds exhibit poor absorption, rapid metabolism, and limited mitochondrial accumulation *in vivo*. These approaches will be essential for distinguishing genuine mitochondrial modulators from compounds whose apparent activities may result from nonspecific bioactivity or assay interference.

Importantly, future investigations should move beyond isolated natural monomers and focus more on classical TCM formulas and herb-pair compatibility strategies, which may exert synergistic multi-target regulatory effects on mitochondrial homeostasis, immune responses, and microbial metabolism. Large-scale randomized clinical trials incorporating mitochondrial biomarkers and precision medicine-based patient stratification will ultimately be necessary to determine the true translational value of TCM-based mitochondrial interventions in IBD.
